# Prevention of Tumor Formation by Latent Gammaherpesvirus Infection

**DOI:** 10.1371/journal.pone.0145678

**Published:** 2015-12-29

**Authors:** S. Raffegerst, B. Steer, M. Hohloch, H. Adler

**Affiliations:** 1 Institute of Molecular Immunology, Helmholtz Zentrum München—German Research Center for Environmental Health (GmbH), Munich, Germany; 2 Research Unit Gene Vectors, Helmholtz Zentrum München—German Research Center for Environmental Health (GmbH), Munich, Germany; Lisbon University, PORTUGAL

## Abstract

Recent reports suggested that chronic herpesvirus infection, as a constituent of the so-called virome, may not only exert harmful effects but may also be beneficial to the host, for example mediating increased resistance to secondary infections or to tumors. To further challenge this concept, specifically regarding increased resistance to tumors, we infected chimeric HLA-DR4-H2-E (DR4) mice, a mouse strain which spontaneously develops hematological tumors, with the rodent herpesvirus murine gammaherpesvirus 68 (MHV-68). Using this model, we observed that infection with wildtype MHV-68 completely prevented tumor formation. This happened, however, at the cost of hyposplenism. In contrast to wildtype infection, infection with a latency-deficient mutant of MHV-68 neither prevented tumor formation nor induced hyposplenism. The underlying mechanisms are not known but might be related to an infection-mediated priming of the immune response, resulting in the suppression of a tumor promoting endogenous retrovirus. Thus, under certain circumstances, chronic herpesvirus infection may prevent the development of tumors.

## Introduction

Herpesviruses are broadly recognized as important pathogens, causing a variety of diseases in humans and in other species. For example, the human gammaherpesviruses Kaposi’s sarcoma-associated herpesvirus (KSHV) and Epstein-Barr virus (EBV) are associated with several tumors: KSHV is associated with lymphoproliferative disorders and Kaposi’s sarcoma [1], and EBV with lymphomas and nasopharyngeal carcinoma [2]. Since herpesviruses establish a lifelong chronic infection and are present in almost every individual, yet cause disease in only a limited number of predisposed individuals, they are considered to be part of the so-called virome [3,4]. This new and emerging concept includes the view that herpesviruses, as constituents of the virome, may not only exert harmful effects but may also be beneficial to the host [4]. Evidence to support this hypothesis comes, for example, from experimental infection of mice with a gammaherpesvirus called murine gammaherpesvirus 68 (MHV-68), which serves as a small animal model to investigate gammaherpesvirus pathogenesis [5]. Using this model, Barton et al. demonstrated that chronic infection of mice with a gammaherpesvirus increased resistance to Listeria monocytogenes and Yersinia pestis [6], and Saito et al. showed that latently infected mice had significantly higher survival to influenza A virus infection due to lower influenza viral loads and decreased lung pathology [7]. In addition, using the same model, White et al. showed that a latent gammaherpesvirus infection armed NK cells, i.e. provided an “arming” event for NK cells, enabling them not only to recognize but also to kill target cells [8]. NK cells armed in this way were able to protect mice against a lethal lymphoma challenge [8], suggesting that chronic herpesvirus infection might perhaps, at least under certain circumstances, also result in increased resistance to tumors. The symbiotic, gammaherpesvirus-induced protection against a subsequent bacterial infection was confirmed by other authors, however, they found the effect to be only transient and concluded that the gammaherpesvirus infection may provide only a temporary benefit [9]. Similarly, one might speculate that the increased resistance to tumors as observed by White et al. [8] was related to the model used and therefore somewhat artificial since latently infected mice were only challenged by intraperitoneal injection with T-cell lymphoma cells (RMA-S).

Therefore, in our study, we wanted to further challenge the hypothesis of potential beneficial effects of a chronic herpesvirus infection, specifically regarding increased resistance to tumors. In contrast to the White et al. [8] studies using an exogenous tumor cell injection model, we employed an endogenous/autochthonous tumor model, whereby chimeric HLA-DR4-H2-E (DR4) mice spontaneously develop diverse hematological malignancies starting around eight months of age [10]. Using this model, we demonstrate that infection with wildtype MHV-68 completely prevented tumor formation, however, at the cost of hyposplenism. In contrast to wildtype infection, infection with a latency-deficient mutant of MHV-68 neither prevented tumor formation nor induced hyposplenism. The underlying mechanisms are not known but might be related to an immune response-mediated interference with a tumor-promoting endogenous retrovirus. Thus, under certain circumstances, chronic herpesvirus infection may prevent the development of tumors.

## Material and Methods

### Cell lines and virus stocks

BHK-21 cells (ATCC CCL-10) were grown in Glasgow-MEM (PAN Biotech, Aidenbach, Germany) supplemented with 5% fetal calf serum (FCS), 5% tryptose phosphate broth, 2 mM L-glutamine, 100 U/mL penicillin and 100 μg/mL streptomycin. NIH3T3 cells (ATCC CRL-1658) were grown in DMEM (Invitrogen, Darmstadt, Germany) supplemented with 10% FCS, 2 mM L-glutamine, 100 U/mL penicillin and 100 μg/mL streptomycin. Working stocks of virus were prepared as previously described [11]. Briefly, stocks were grown by infecting BHK-21 cells. After showing complete cytopathic effect (CPE), BHK-21 cells were harvested and the supernatant was used as working stock after two times freezing/thawing the cells and removing cell debris by centrifugation. Virus titers were determined by plaque assays. Briefly, 10-fold dilutions were incubated on BHK-21 cells for 90 min at 37°C. After removing the inoculum, cells were incubated for 5 days at 37°C with fresh medium containing methylcellulose. Cells were stained with 0.1% crystal violet solution to determine the number of plaques.

### Infection of mice

HLA-DR4-H2-E (DR4) [12] mice were bred and propagated under SPF conditions at the Helmholtz Zentrum München. During the MHV-68 infection period, mice were housed in individually ventilated cages (IVC). Mice were infected intranasally (i.n.) at an age of 10–13 weeks with 5x10^4^ plaque forming units (PFU) of wt or latency-deficient MHV-68 (ORF73 deletion mutant [Δ73]). Δ73 was constructed by ET-cloning as previously described [11]. Briefly, a part of ORF73 was first replaced with a tetracycline (Tet) resistance gene flanked by FRT sites. Subsequently, the Tet resistance cassette was removed by FLP-mediated recombination, resulting in a deletion of nucleotides 104141 to 104594. Δ73 was characterized by restriction enzyme analysis with several restriction enzymes and by sequencing across the mutated region. Prior to i.n. infection, mice were anesthetized with ketamine and xylazine. All animal experiments were in compliance with protocols approved by the local Animal Care and Use Committee (District Government of Upper Bavaria; permit number 124/08). Mice were monitored daily for signs of disease, and any mice that appeared moribund were sacrificed by exposure to CO_2_. Then, a detailed inspection for signs of tumor development, in particular for the size of the spleen, was performed, and samples were taken for further analyses.

### Flow cytometry

Surface marker expression was analyzed using the following antibodies: CD3 (clone 17A2) and B220 (clone RA3-6B2) (all from eBiocience according to data sheet). Staining was performed in the presence of Fc-receptor blocking antibody (clone 2.4G8, a kind gift of E. Kremmer, Helmholtz Zentrum München). Intracellular cytokine staining for IFN-ɣ (eBioscience Clone XMG1.2) was performed as follows: 1x10^6^ cells/ well were put in 200μL of stimulation media (RPMI 1640 + 10% FCS supplemented with PMA (20ng/mL), Ionomycin (1μg/mL) and Brefeldin A (10μg/mL)) in a 96 well flat bottom plate and incubated for 4h at 37°C. Unstimulated cells were handled in parallel, without PMA/Iono stimulation but with Brefeldin A. After incubation, cells were washed twice with PBS and used for surface marker staining (B220) followed by intracellular CD3 and IFN-ɣ staining with Fix/Perm-Kit (eBioscience) as described by the manufacturer.

All cells were processed on a LSRII Fortessa Flow Cytometer (BD) and analyzed with FlowJo9.6.2 software. Dead cells were excluded using Live/Dead Fixable Blue Dead Cell Stain Kit (Invitrogen) and doublets by gating on single cells.

### Determination of env copy numbers

For quantitative detection of Ecotropic env gene, we designed a plasmid standard for the env gene as well as the 18s rRNA reference gene for normalization. Env copy numbers were determined by quantitative PCR (qPCR) on a Light Cycler instrument (LC2.0 Roche). Before, the env gene and the reference 18s rRNA gene were each cloned in the backbone of a PGEM gene expression vector (Promega). By digestion of amplicons and vector with HindIII and EcoRI restriction enzymes (NEB), amplicons could be ligated overnight in the digested vector backbones. Then, the copy numbers of env and 18s rRNA could be calculated and titrated accordingly to produce a copy number standard for qPCR for both genes. Oligos for cloning were as follows: 5’env HindIII: ATAAAGCTTATGGCGCGTTCAACGCTCTC; 3’ env EcoRI: ATAGAATTCCTATGGCTCGTACTCTATAGG; 5‘ 18s HindIII: ATAAAGCTTAAGCTTCGGCTACCACATCCAAG; 3’ 18s EcoRI: ATAGAATTCGCTGGAATTACCGCGGCTGCTG. Primer for amplification of env: 5’ CACCCTCTGTGGACCTGGTG; 3’ TAGCTTGAGTCTGTTCCAGGC. Primer for amplification of 18s rRNA: 5’ AGCTTCGGCTACCACATCCAAG, 3’ GCTGGAATTACCGCGGCTGCTG.

RNA from splenocytes was isolated with the RNeasy Mini Kit from Qiagen according to manufacturer’s protocol and eluted in pure grade H_2_O. For first strand cDNA synthesis, 1000ng total RNA were reverse transcribed with the affinity script first strand cDNA synthesis kit (Stratagene) with oligo (dT) primers and MMLV reverse transcriptase in 20*μ*L volume as described by the manufacturer. 1*μ*L of cDNA was used as template for qPCR with FastStart SYBR Green Master Mix Kit (Roche) as described by the manufacturer. The conditions were as follows: initial denaturation at 95°C for 10 min, followed by 30 amplification cycles (denaturation: 95°C for 10 sec; annealing: 57°C for 25 sec; elongation: 72°C for 10 sec), followed by melting curve analysis.

### Statistical methods

If not otherwise indicated, data were analyzed by two-tailed, unpaired Student’s t-test.

## Results

### Infection of DR4 mice with wildtype (wt) MHV-68 prevents tumor development

**Fig 1:**
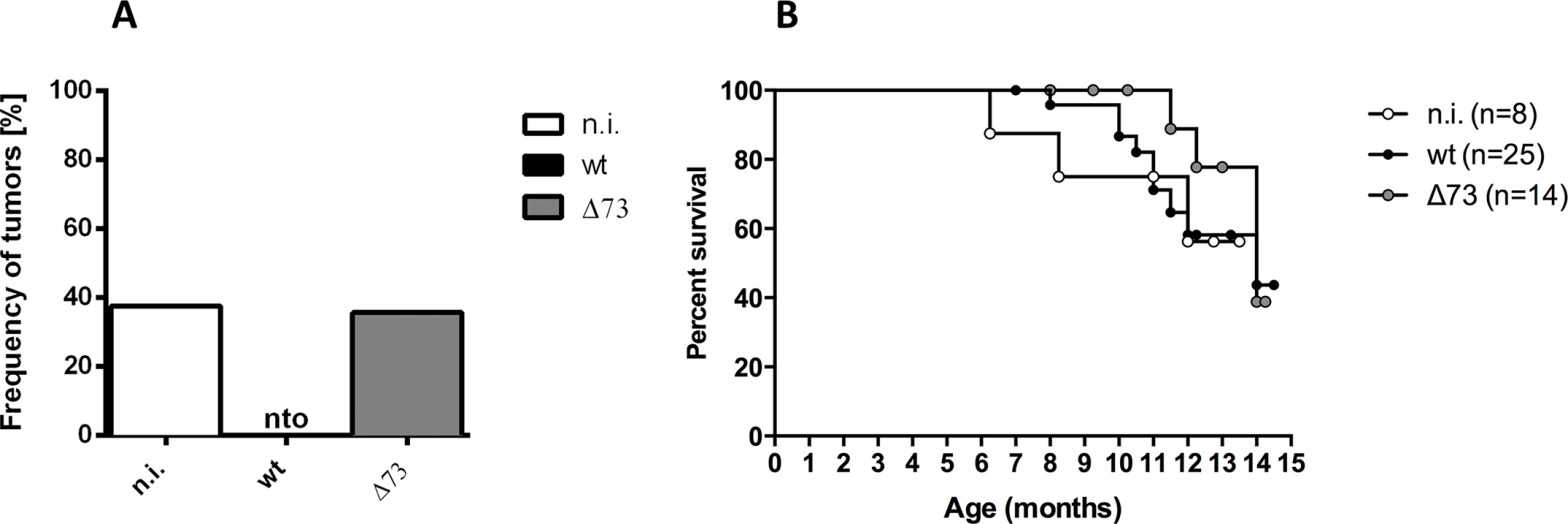
Infection of DR4 mice with wt MHV-68 prevents tumor development but does not improve overall survival. A) Frequency of tumors. B) Overall survival. Mice were infected at an age of 10–13 weeks and monitored up to an age of 15 months. n.i.: not-infected; wt: infected with wt MHV-68; Δ73: infected with latency-deficient MHV-68, carrying a deletion in ORF73; nto: no tumors observed; numbers in brackets (n): number of mice analysed.

To address the question whether a chronic herpesvirus infection might be associated with an increased resistance to tumors, we used chimeric HLA-DR4-H2-E (DR4) mice which spontaneously develop diverse hematological tumors [10]. DR4 mice were left uninfected or were infected with wt or latency-deficient (Δ73) MHV-68. Δ73 MHV-68 carries a deletion in ORF73. ORF73 of MHV-68 has been shown to be dispensable for lytic replication but to be critical for the establishment of latency [13,14]. When we analyzed the mice with regard to the development of tumors [10], we observed that infection with wt MHV-68 completely prevented tumor formation (Fig 1A). In contrast, uninfected mice and mice infected with Δ73 MHV-68 displayed a frequency of tumors of 37.5% and 35.7%, respectively. This was consistent with the tumor frequency at this age of the mice, as observed in our previous study [10]. We concluded from these data that infection of DR4 mice with MHV-68 can prevent the formation of tumors. The establishment of latency is required for this effect since wt and Δ73 MHV-68 undergo comparable productive acute replication but only wt and not Δ73 MHV-68 can establish a chronic, latent infection.

### Uninfected DR4 mice and DR4 mice infected with wt or Δ73 MHV-68 display similar overall survival

Surprisingly, when we analyzed survival of mice within the observation period (up to the age of 15 months), we found that the overall survival was comparable among all groups of mice (Fig 1B). Thus, prevention of tumor formation by infection with wt MHV-68 did not result in an increased survival rate, suggesting that the potential benefit (prevention of tumor formation) might be nullified by hitherto unknown additional effects leading to fatal disease not related to tumor development.

### Infection of DR4 mice with wt MHV-68 results in hypospleny

**Fig 2:**
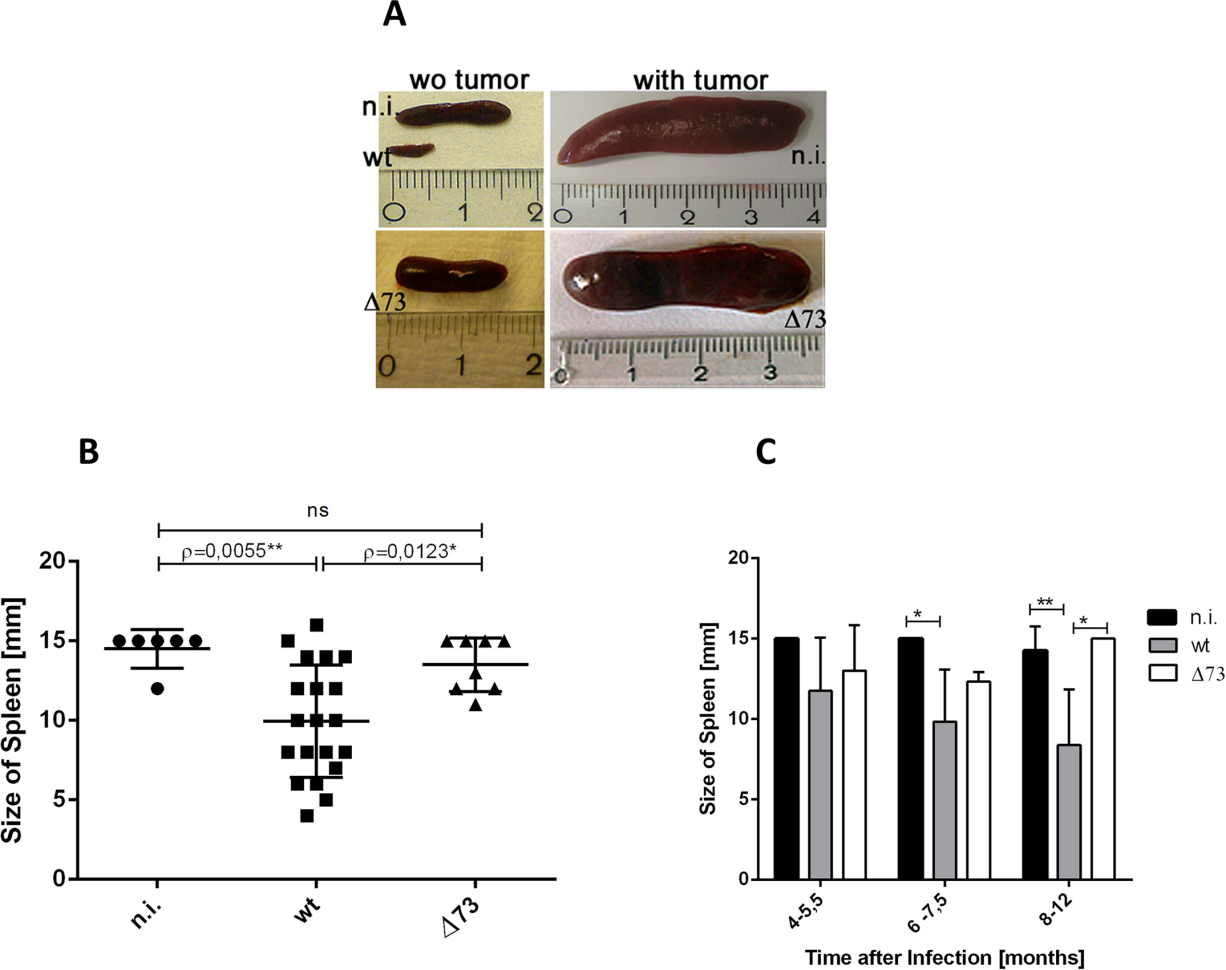
Infection of DR4 mice with wt MHV-68 results in hypospleny. A) Photomicrographs showing examples of normal spleens, of a very tiny spleen and of tumor spleens. B) Quantitative analysis of hyposplenism. Mice were infected at an age of 10–13 weeks. The spleen sizes of all individual mice, independent of the time point of analysis, are summarized for each group. An analysis over time after infection is shown in panel C. Each symbol represents an individual mouse, and the means ± SD are shown. Spleens with tumors were excluded from this analysis. C) Analysis of hyposplenism over time after infection. Spleens with tumors were excluded from this analysis. Means + SD of the following numbers of mice are shown: 4–5,5 months after infection: n.i. (n = 2), wt (n = 4), Δ73 (n = 2); 6–7,5 months after infection: n.i. (n = 2), wt (n = 6), Δ73 (n = 3); 8–12 months after infection: n.i. (n = 2), wt (n = 9), Δ73 (n = 3). n.i.: not-infected; wt: infected with wt MHV-68; Δ73: infected with latency-deficient MHV-68, carrying a deletion in ORF73; wo tumor: without tumor; *: P = 0.032; **: P = 0.009.

A detailed analysis revealed that uninfected, non-diseased DR4 mice showed normal spleens while uninfected, diseased mice showed splenomegaly, indicating tumor development. Comparable observations were made in mice infected with Δ73 MHV-68. In sharp contrast, very small spleens were observed in most mice infected with wt MHV-68. Examples of these findings are shown in Fig 2A, and a quantitative analysis is provided in Fig 2B. The hypospleny after infection with wt MHV-68 became apparent at approximately 5 months after infection, reached significance at 6 months post infection, and was even further pronounced at later time points (Fig 2C).

### Infection of DR4 mice with wt MHV-68 results in lower copy numbers of an endogenous retrovirus

**Fig 3:**
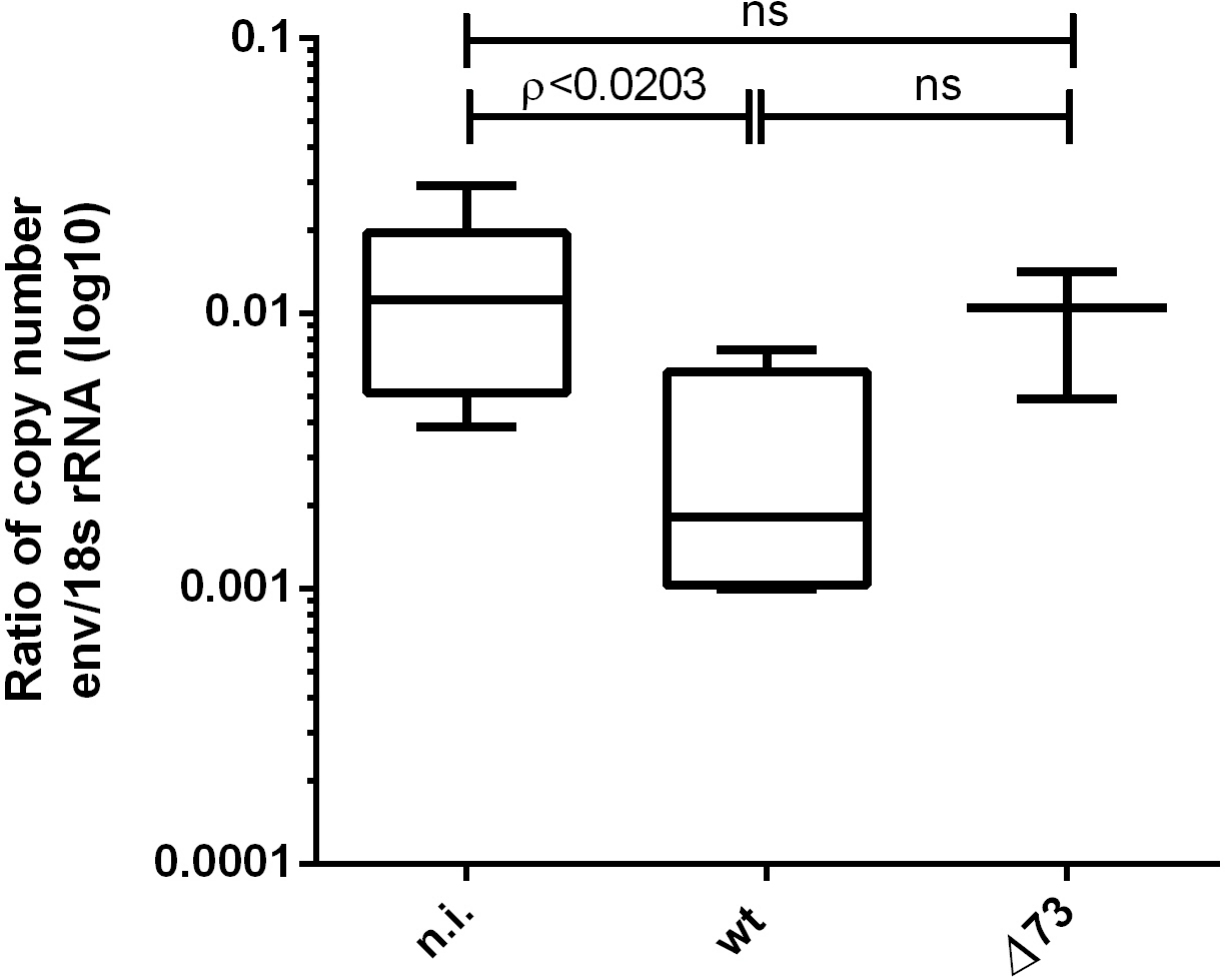
Infection of DR4 mice with wt MHV-68 reduces the copy number of an endogenous retrovirus. The copy number of the endogenous retrovirus was determined by RT-qPCR and is presented as env-copy number relative to the copy number of 18s rRNA. Mice were infected at an age of 10–13 weeks, and the env-copy number was determined 6–9 months after infection. n.i.: not-infected (n = 6); wt: infected with wt MHV-68 (n = 4); Δ73: infected with latency-deficient MHV-68, carrying a deletion in ORF73 (n = 3).

In the DR4 mouse model, activation of an endogenous retrovirus contributes to the development of lymphoid tumors (Raffegerst et al., manuscript in preparation). Therefore, and to gain insight into how infection with wt MHV-68 might interfere with tumor formation in this mouse model, we investigated whether infection with MHV-68 might influence the copy number of the endogenous retrovirus. To this end, the copy numbers were determined in sera of uninfected DR4 mice and of mice infected with either wt or Δ73 MHV-68. As shown in Fig 3, infection with wt MHV-68 resulted in a significant reduction in the copy number of the endogenous retrovirus when compared to uninfected mice. In contrast, infection with Δ73 MHV-68 did not result in a reduction in the copy number of the endogenous retrovirus, again suggesting that the establishment of a latent infection is required for this effect.

### Infection of DR4 mice with wt MHV-68 primes T cells for enhanced interferon-γ production

**Fig 4:**
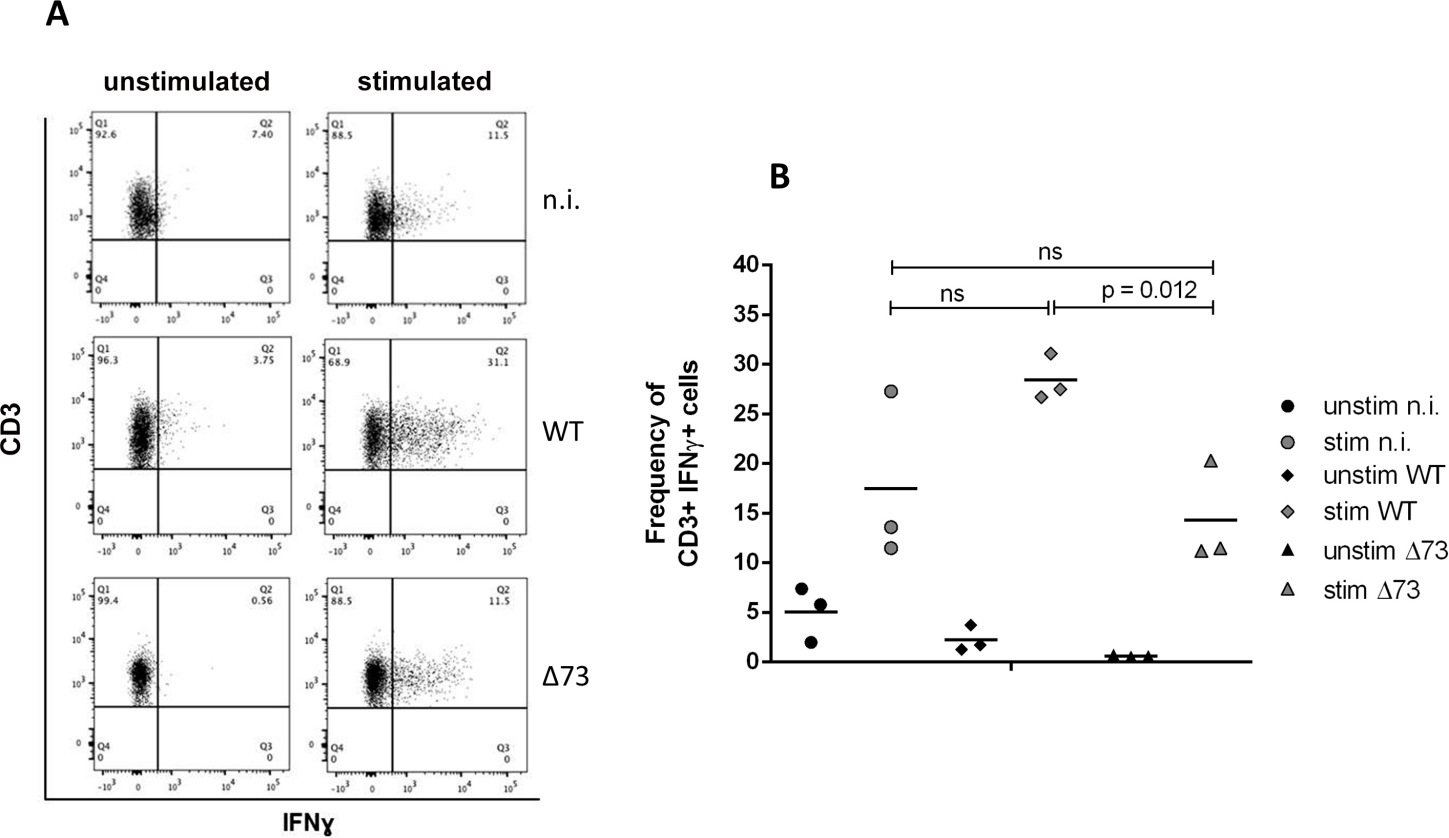
Infection of DR4 mice with wt MHV-68 primes T cells for enhanced interferon-γ production. Mice were infected at an age of 10–13 weeks. Splenocytes from uninfected and infected DR4 mice were isolated 8–11.75 months after infection, re-stimulated with PMA/ionomycin (stim) or left unstimulated (unstim), and were subsequently analyzed by multicolor FACS analysis for intracellular IFN-γ production. A) Representative Dot Plots of one mouse per group are shown. B) Summary of three mice per group. Each symbol represents an individual mouse, and the bars represent the mean. n.i.: not-infected (n = 3); wt: infected with wt MHV-68 (n = 3); Δ73: infected with latency-deficient MHV-68, carrying a deletion in ORF73 (n = 3).

Since White et al. [8] had shown that latent MHV-68 infection can arm NK cells, resulting in an increased capacity to produce IFN-γ, we were interested whether a similar phenomenon might occur in our DR4 model. For this purpose, we isolated splenocytes from uninfected DR4 mice and from mice infected with either wt or Δ73 MHV-68, re-stimulated them with PMA/ionomycin and subsequently analyzed various cell types by multicolor FACS analysis for intracellular IFN-γ production. Infection with wt MHV-68 strongly enhanced the capacity of CD3-positive T cells to produce IFN-γ, while infection with Δ73 MHV-68 did not (Fig 4). No differences between cells isolated from infected or uninfected mice were observed for NK cells (data not shown). Thus, activation of T cells by wt MHV-68 infection might be a mechanism that contributes to prevention of tumor formation, perhaps leading to an impact on the endogenous retrovirus virome.

## Discussion

It has been proposed that herpesviruses are part of the virome and as such, they may cause disease in some situations but also be beneficial to the host in other instances [4]. Our data support the hypothesis of potential beneficial effects of a chronic herpesvirus infection, specifically regarding increased resistance to tumors. Using chimeric HLA-DR4-H2-E (DR4) mice that spontaneously develop diverse hematological malignancies starting around eight months of age, we demonstrate that infection with wt MHV-68 completely prevented tumor formation, albeit at the cost of hyposplenism. In contrast to infection with wt virus, infection with a latency-deficient mutant of MHV-68 neither prevented tumor formation nor induced hyposplenism. wt MHV-68 is able to establish and to sporadically reactivate from latency while the ORF73-deficient mutant of MHV-68 is unable to establish latency [13,14], indicating that a latent infection was necessary for the observed effects. We observed that MHV-68 infection primed T cells for enhanced IFN-gamma production upon secondary stimulation. MHV-68 infection also led to a reduction of the copy numbers of an endogenous, tumor-promoting retrovirus. However, whether it is the reduction of endogenous retrovirus copy load which impeded tumor formation is currently not known. Nevertheless, such a scenario is conceivable since there are studies showing interference between herpesviruses and retroviruses. For example, human herpesvirus 7 can suppress HIV replication via modulation of CD4 [15,16]. Furthermore, the terminal membrane proteins of herpesvirus saimiri, a gammaheresvirus closely related to MHV-68, were proposed to have the ability to modulate the replication of competing retroviruses [17]. Interestingly, Yu et al. [18] recently showed a pivotal role for Toll-like receptors, in particular TLR3, TLR7 and TLR9, in the immune control of endogenous retrovirus-induced tumors. Notably, we have previously shown that MHV-68 interacts with TLR9 [19]. Thus, it seems possible that in our model, the prevention of tumor formation is caused by interference of MHV-68 with the endogenous retrovirus at multiple steps.

Our observation of T cell activation is similar to the findings of White et al. who showed that latent MHV-68 infection provides an “arming” event for NK cells, enabling them to kill tumor cells [8]. In our model, we did not observe differences between NK cells isolated from MHV-68 infected and uninfected DR4 mice (data not shown).

Why infection with wt virus resulted in hyposplenism is not clear, however, it might be related to specific genetic alterations present in homozygous DR4 mice, since it was not observed in an F1 generation of mice obtained by crossing DR4 mice with C57BL/6 mice (data not shown). Obviously, the genetic constitution can contribute to hyposplenism after MHV-68 infection since it has also been described for IFN-gamma-receptor deficient mice [20].

It has been suggested that there is a complex and tightly regulated balance between the immunologic benefit and the immunologic harm caused by chronic herpesvirus infections [3]. Harmless infections may play a role in shaping the normal immune response, however, at the cost of inducing disease in situations where the immune system is altered [3]. Consistent with this hypothesis are studies describing either a beneficial or a detrimental influence of a chronic MHV-68 infection on the development of diseases triggered by other causes. For example, latent MHV-68 infection protected lupus-prone mice from the development of autoimmunity [21] while it exacerbated metastatic disease in a mouse mammary tumor model [22]. Similar observations have also been made in humans. While EBV infection can cause lymphomas and nasopharyngeal carcinoma, controlled EBV reactivation in the setting of hematopoietic stem cell transplantation was associated with improved survival, presumably because of a significant increase in circulating NK cells [23]. Furthermore, early-life EBV infection seems to provide a protective effect against the development of Th2-mediated pathologies [5,24].

In summary, our data suggest that chronic herpesvirus infection may prevent the development of tumors, at least under certain circumstances.

## References

[1] Schulz TF. Kaposi's sarcoma-associated herpesvirus (human herpesvirus-8). J Gen Virol. 1998; 79:1573–1591. 9680119 10.1099/0022-1317-79-7-1573

[2] Rickinson AB, Kieff E. Epstein-Barr Virus In: Knipe DM, Howley PM, Griffin DE, Martin MA, Lamb RA et al, editors. Fields—Virology. Philadelphia: Lippincott Williams & Wilkins 2001; pp. 2575–2627.

[3] Virgin HW, Wherry EJ, Ahmed R. Redefining chronic viral infection. Cell. 2009; 138:30–50. 10.1016/j.cell.2009.06.036 19596234

[4] Virgin HW. The virome in mammalian physiology and disease. Cell. 2014; 157:142–150. 10.1016/j.cell.2014.02.032 24679532 PMC3977141

[5] Barton E, Mandal P, Speck SH. Pathogenesis and Host Control of Gammaherpesviruses: Lessons from the Mouse. Annu Rev Immunol. 2011; 29:351–397. 10.1146/annurev-immunol-072710-081639 21219186

[6] Barton ES, White DW, Cathelyn JS, Brett-McClellan KA, Engle M, Diamond MS et al Herpesvirus latency confers symbiotic protection from bacterial infection. Nature. 2007; 447:326–329. 17507983 10.1038/nature05762

[7] Saito F, Ito T, Connett JM, Schaller MA, Carson WF, Hogaboam CM et al MHV68 latency modulates the host immune response to influenza A virus. Inflammation. 2013; 36:1295–1303. 10.1007/s10753-013-9668-1 23807051 PMC3825492

[8] White DW, Keppel CR, Schneider SE, Reese TA, Coder J, Payton JE et al Latent herpesvirus infection arms NK cells. Blood. 2010; 115:4377–4383. 10.1182/blood-2009-09-245464 20139098 PMC2881492

[9] Yager EJ, Szaba FM, Kummer LW, Lanzer KG, Burkum CE, Smiley ST et al gamma-Herpesvirus-induced protection against bacterial infection is transient. Viral Immunol. 2009; 22:67–72. 10.1089/vim.2008.0086 19210230 PMC2952138

[10] Raffegerst SH, Hoelzlwimmer G, Kunder S, Mysliwietz J, Quintanilla-Martinez L, Schendel DJ. Diverse hematological malignancies including Hodgkin-like lymphomas develop in chimeric MHC class II transgenic mice. PLoS ONE. 2009; 4:e8539 10.1371/journal.pone.0008539 20046882 PMC2796171

[11] Adler H, Messerle M, Wagner M, Koszinowski UH. Cloning and mutagenesis of the murine gammaherpesvirus 68 genome as an infectious bacterial artificial chromosome. J Virol. 2000; 74:6964–6974. 10888635 10.1128/jvi.74.15.6964-6974.2000 PMC112213

[12] Ito K, Bian HJ, Molina M, Han J, Magram J, Saar E et al HLA-DR4-IE chimeric class II transgenic, murine class II-deficient mice are susceptible to experimental allergic encephalomyelitis. J Exp Med. 1996; 183:2635–2644. 8676084 10.1084/jem.183.6.2635 PMC2192625

[13] Fowler P, Marques S, Simas JP, Efstathiou S. ORF73 of murine herpesvirus-68 is critical for the establishment and maintenance of latency. J Gen Virol. 2003; 84:3405–3416. 14645921 10.1099/vir.0.19594-0

[14] Moorman NJ, Willer DO, Speck SH. The gammaherpesvirus 68 latency-associated nuclear antigen homolog is critical for the establishment of splenic latency. J Virol. 2003; 77:10295–10303. 12970414 10.1128/JVI.77.19.10295-10303.2003 PMC228443

[15] Lisco A, Grivel JC, Biancotto A, Vanpouille C, Origgi F, Malnati MS et al Viral interactions in human lymphoid tissue: Human herpesvirus 7 suppresses the replication of CCR5-tropic human immunodeficiency virus type 1 via CD4 modulation. J Virol. 2007; 81:708–717. 17065205 10.1128/JVI.01367-06 PMC1797468

[16] Crowley RW, Secchiero P, Zella D, Cara A, Gallo RC, Lusso P. Interference between human herpesvirus 7 and HIV-1 in mononuclear phagocytes. J Immunol. 1996; 156:2004–2008. 8596056

[17] Raymond AD, Hasham M, Tsygankov AY, Henderson EE. Herpesvirus saimiri terminal membrane proteins modulate HIV-1 replication by altering Nef and Tat functions. Curr HIV Res. 2007; 5:79–86. 17266559 10.2174/157016207779316314

[18] Yu P, Lubben W, Slomka H, Gebler J, Konert M, Cai C et al Nucleic acid-sensing Toll-like receptors are essential for the control of endogenous retrovirus viremia and ERV-induced tumors. Immunity. 2012; 37:867–879. 10.1016/j.immuni.2012.07.018 23142781

[19] Guggemoos S, Hangel D, Hamm S, Heit A, Bauer S, Adler H. TLR9 Contributes to Antiviral Immunity during Gammaherpesvirus Infection. J Immunol. 2008; 180:438–443. 18097045 10.4049/jimmunol.180.1.438

[20] Ebrahimi B, Dutia BM, Brownstein DG, Nash AA. Murine gammaherpesvirus-68 infection causes multi-organ fibrosis and alters leukocyte trafficking in interferon-ɣ receptor knockout mice. Am J Pathol. 2001; 158:2117–2125. 11395389 10.1016/s0002-9440(10)64683-4 PMC1892003

[21] Larson JD, Thurman JM, Rubtsov AV, Claypool D, Marrack P, Van Dyk LF et al Murine gammaherpesvirus 68 infection protects lupus-prone mice from the development of autoimmunity. Proc Natl Acad Sci U S A. 2012; 109:E1092–E1100. 10.1073/pnas.1203019109 22474381 PMC3345002

[22] Chauhan VS, Nelson DA, Roy LD, Mukherjee P, Bost KL. Exacerbated metastatic disease in a mouse mammary tumor model following latent gammaherpesvirus infection. Infect Agent Cancer. 2012; 7:11–17. 10.1186/1750-9378-7-11 22642913 PMC3565933

[23] Auger S, Orsini M, Ceballos P, Fegueux N, Kanouni T, Caumes B et al Controlled Epstein-Barr virus reactivation after allogeneic transplantation is associated with improved survival. Eur J Haematol. 2014; 92:421–428. 10.1111/ejh.12260 24400833

[24] Saghafian-Hedengren S, Sverremark-Ekstrom E, Linde A, Lilja G, Nilsson C. Early-life EBV infection protects against persistent IgE sensitization. J Allergy Clin Immunol. 2010; 125:433–438. 10.1016/j.jaci.2009.09.033 19963258

